# Interference of COVID-19 Vaccination With PET/CT Leads to Unnecessary Additional Imaging in a Patient With Metastatic Cutaneous Melanoma—Case Report

**DOI:** 10.3389/fonc.2021.690443

**Published:** 2021-08-03

**Authors:** Rafał Czepczyński, Jolanta Szczurek, Jacek Mackiewicz, Marek Ruchała

**Affiliations:** ^1^Department of Endocrinology, Metabolism and Internal Diseases, Poznan University of Medical Sciences, Poznań, Poland; ^2^Department of Nuclear Medicine, Affidea, Poznań, Poland; ^3^Department of Medical and Experimental Oncology, Poznan University of Medical Sciences, Poznań, Poland

**Keywords:** malignant melanoma, PET/CT, COVID-19, vaccination, metastases

## Abstract

The COVID-19 pandemic has widely influenced oncological imaging mainly by presenting unexpected pulmonary and mediastinal lesions. The ongoing global program of vaccination has led to incidental diagnosis of axillary lymphadenopathy. We present a case of increased accumulation of ^18^F-FDG in an axillary lymph node in a PET/CT scan performed in a 43-year-old female patient with metastatic melanoma. The scan was performed 4 days after the AZD1222 vaccination. The occurrence of lymphadenopathy was verified with another PET/CT scan scheduled one month later. This case report presents a possible misinterpretation of PET/CT images caused by the recent COVID-19 vaccination. To avoid distress of the patient and unnecessary oncological diagnostics to verify the findings, we recommend avoiding scheduling PET/CT shortly after vaccination.

## Introduction

Positron emission tomography with computed tomography (PET/CT) using ^18^F-fluoro-deoxyglucose (^18^F-FDG) is a valuable tool used to monitor treatment of melanoma, especially its metastatic forms subjected to immunotherapy (1). In stage III cutaneous melanoma, sensitivity in detecting distant metastases during follow-up ranges between 82 and 100%, and the specificity ranges between 45 and 100% (2). With regard to lymph node metastases, PET/CT shows sensitivity of 91% for nodes >10 mm and 69% for smaller nodes (with a similar specificity of 71%) (3). The inflammatory reaction of the lymph nodes is one of the main causes of the false positive PET/CT findings in oncological patients. A non-specific nodal ^18^F-FDG uptake may lead to a false diagnosis of metastases and to the initiation of an unnecessary treatment.

The widespread COVID-19 vaccination has raised a lot of questions with regard to its potential complications and side-effects. Many patients experience local pain in the injection site; some of them suffer from generalized inflammatory reactions, including fever and fatigue (4). As it has been recently shown, local inflammatory reaction in the lymphatic system may have potential implications for imaging. The vaccine-induced lymphadenopathy may also pose a challenge in the PET/CT interpretation (5). In this paper, we report a patient with stage IV melanoma who had a PET/CT performed incidentally few days after COVID-19 vaccination that resulted in a false positive finding in an axillary lymph node.

## Case Presentation

A 43-year-old female with the diagnosis of metastatic melanoma treated with nivolumab was reported for a ^18^F-FDG PET/CT scan to exclude disease progression shortly after COVID-19 vaccination.

**Figure 1:**
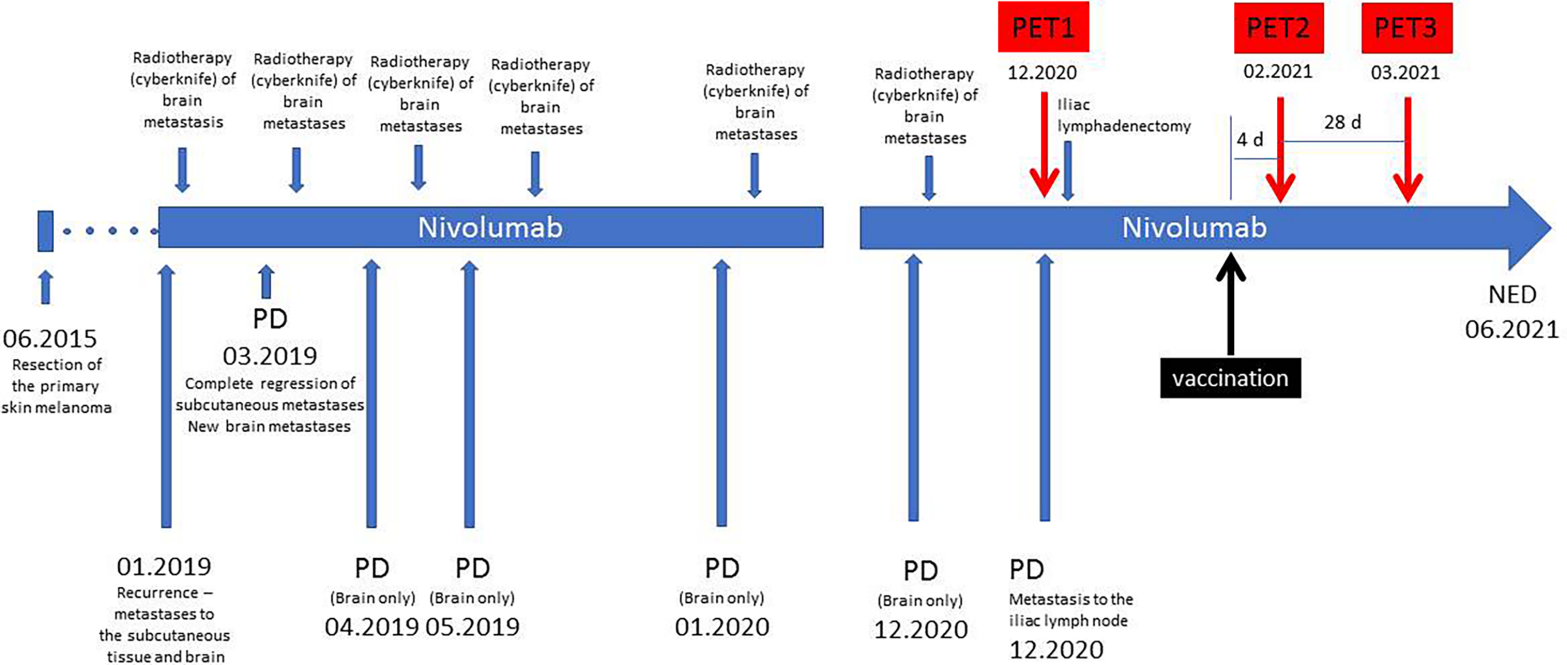
Timeline of the treatment. PD, progression of the disease; NED, no evidence of the disease.

The timeline of the history of the patient is presented in **Figure 1**. She was diagnosed of a primary cutaneous melanoma of the right thigh in June 2015. The patient was previously healthy, with no history of other malignancies, surgery, or medication. There was no personal or family history of melanoma. The lesion was removed, and the final diagnosis was: cutaneous melanoma, *BRAF* wild-type, pT2aN0M0. In January 2019, a recurrence of the disease in form of the subcutaneous and brain metastases was diagnosed with the use of CT. A single cerebral metastasis was confirmed by MRI. After two weeks, nivolumab treatment was initiated, with a dose of 480 mg every 4 weeks. After the first dose of nivolumab, the cyberknife radiotherapy of the brain metastasis was performed. After two months of systemic treatment, all subcutaneous metastases disappeared; however six new brain metastases were detected in another MRI. All these new lesions were subsequently treated with cyberknife. The patient continued the nivolumab therapy beyond progression. Thereafter, during nivolumab treatment, she developed a further disease progression in the brain (04.2019, 05.2019, 01.2020, 12.2020). With each progression, one or two new brain metastases were found in the MRI. These lesions did not exceed 1 cm and were asymptomatic. After each occurrence, the cerebral metastases were treated with cyberknife. All extracerebral metastases were still in regression until December 2020 when some metabolically active lymph nodes in the right iliac region were detected in a PET/CT scan (PET1). In January 2021, a robot-assisted right iliac lymphadenectomy was performed, and the metastatic character of the iliac lymph nodes was histologically confirmed.

**Figure 2:**
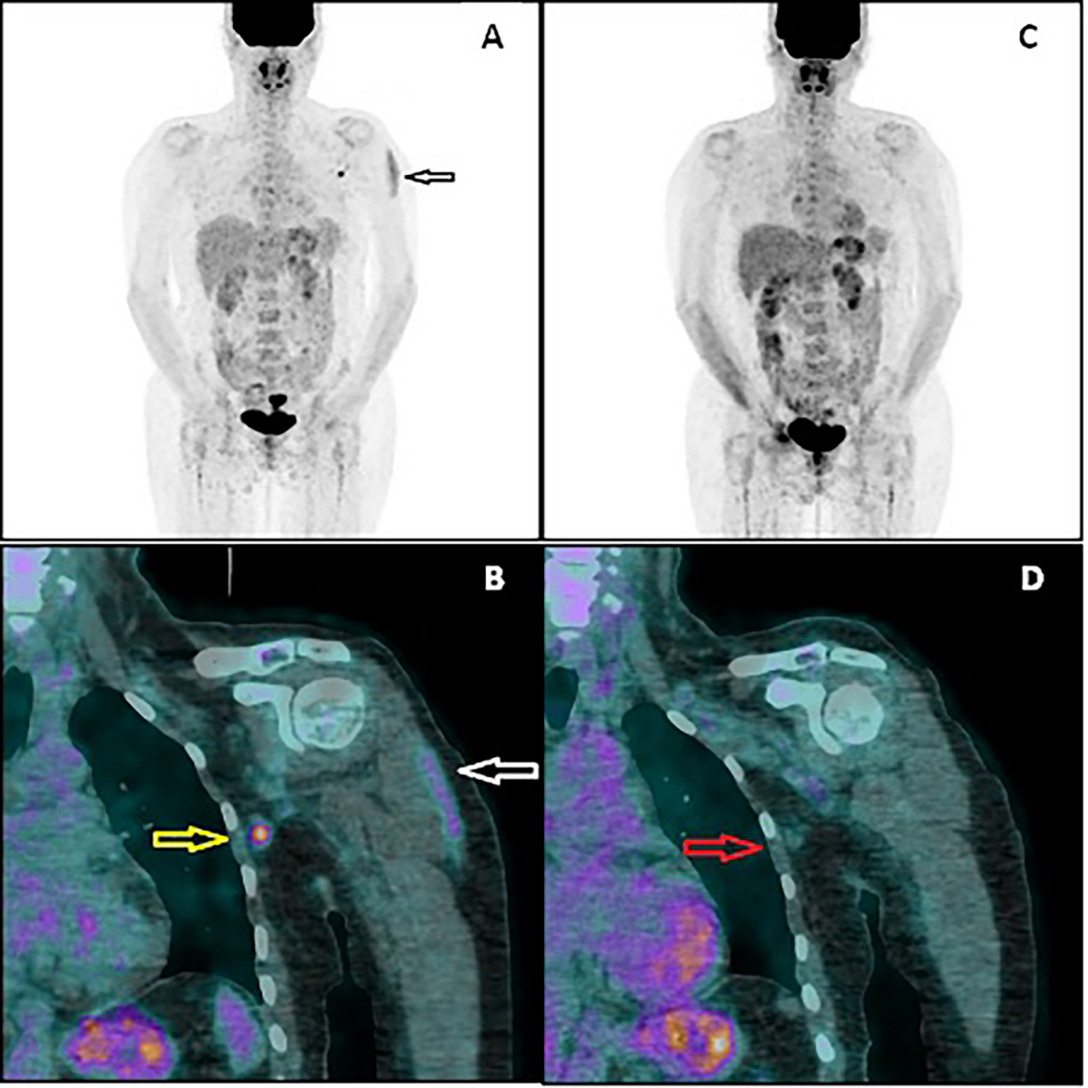
PET/CT image performed 4 days after vaccination **(A, B)**. The multiple-intensity projection image **(A)** showing increased ^18^F-FDG uptake in the left deltoid muscle (black arrow) and in the left axillary region. Fused coronal image **(B)** showing the uptake in the muscle (white arrow) and in the axillary lymph node (yellow arrow). PET/CT image performed 32 days after vaccination **(C, D)**. Both the muscular uptake and nodal uptake have disappeared. The referred axillary lymph node (red arrow) shows similar morphology but no ^18^F-FDG accumulation.

In February 2021, the patient underwent AZD1222 COVID-19 vaccination (first dose injected into her left arm). Four days later, another PET/CT was performed to exclude new extracerebral metastases (PET2). No sign of melanoma recurrence was found in the iliac lymph nodes or central nervous system. However, a metabolically active lymph node in the left axillary region was noted (**Figure 2**). The lymph node had the dimension of 9 × 7 mm, and the maximal standardized uptake value was 5.2. Additionally, an area of increased ^18^F-FDG accumulation was found in the left deltoid muscle that corresponded to the site of the recent vaccination. An inflammatory reaction to the injection was suspected to be responsible for the ^18^F-FDG accumulation in the axillary lymph node. However, in order to rule out a melanoma metastasis in the axillary lymph node, a follow-up PET/CT (PET3) was recommended 28 days later (March 2021, 32 days after vaccination). This scan did not present any ^18^F-FDG accumulation in the reported lymph node. The diameter of the node did not change. No other finding was reported, except for a focus of slightly increased ^18^F-FDG accumulation (diameter of 10 mm) in the right cerebellar lobe that had not been present in the PET2 scan. Fortunately, the subsequent MRI did not confirm any lesion in the cerebellum and did not show any other intracranial recurrence. However, further MRI monitoring of the central nervous system has been recommended.

To date (June, 2021), the patient does not present any active metastases (NED—no evidence of disease). Moreover, her performance status remains WHO 0 from the initial diagnosis until now. The treatment beyond disease progression was beneficial to the patient. In addition, at each disease progression, the patient was offered a second line treatment with ipilimumab, to which the patient did not consent due to its high toxicity and low efficacy (6). The patient was informed that there was insufficient evidence for the treatment with nivolumab beyond confirmed progression (7). Due to the fact that the patient did not consent to the ipilimumab treatment, as well as to the lack of a clinical trial, the continuation of nivolumab therapy was the only reasonable treatment option. In conclusion, the continued treatment beyond progression was decided due to the low tumor burden, the motivation of the patient and good performance status and, the lack of other treatment options.

The patient gave consent for the publication of her case.

## Discussion

PET/CT is an established imaging modality used in oncology on an every-day basis. It is well-known that foci of non-oncological pathology can accumulate ^18^F-FDG similarly to the malignant tumors, nodal and distant metastases. The examples of such benign, metabolically active lesions include pulmonary tuberculosis, benign thyroid nodules, diverticulitis, *etc.* (8–10). Also, reactive lymph nodes can present as metabolically active, mimicking nodal metastases (11). Common situations like that include cervical lymphadenopathy after infection of the upper respiratory tract or tonsillitis, mediastinal lymphadenopathy in case of pneumonia or sarcoidosis, and inguinal lymph node metabolic stimulation due to a lower extremity injury. Careful anamnesis prior to the scan, not excluding apparently irrelevant conditions, like tooth pain or transient fever, may prevent a misinterpretation of the images.

In the every-day practice of a PET/CT department, the occurrence of vaccination-induced lymphadenopathy is a new phenomenon. Several authors have already reported the unexpected findings of increased ^18^F-FDG accumulation in the axillary lymph nodes (5, 12–14). It may cause serious doubts regarding the character of lymphadenopathy in cases of melanoma and other malignancies with an aggressive dissemination pattern. The presented patient had a history of lymph node metastases in the inguinal region that was obviously correlated with the primary location in the ipsilateral lower extremity. However, the metastatic behavior of melanoma can be unpredictable, and a metastasis in the contralateral axillary fossa could not be excluded, especially when knowing that the progression of the disease with new brain metastases during systemic treatment had occurred several times. The coexistence of all these risk factors has led to the recommendation of an early follow-up scan (PET3). Although the axillary metastasis has been excluded by the negative PET/CT, an increased caution of the reporting physician, who was aware of the history of the patient, led to another false positive finding—the cerebellar focus suspected of being another recurrence in the central nervous system. The reporting attention of the physician to possible intracranial foci was alerted because of the well-known low sensitivity of ^18^F-FDG PET/CT in the detection of brain metastases due to the physiological radionuclide uptake in the gray matter. The rapid application of MR has led to the exclusion of relapse.

Worldwide COVID-19 vaccination is an unprecedented program of the medical interventions performed on an enormous global population in a relatively short time (4). What is more, commonly, the intramuscular vaccine injection is performed twice in each subject. Also oncological patients, referred to a PET/CT scan as a part of their routine management, are independently vaccinated and the schedules of the vaccination and imaging are not always coordinated, as they are being organized by separate institutions. This may lead to a situation of equivocal PET/CT findings as presented in this case report.

Interestingly, the sign of elevated ^18^F-FDG accumulation does not occur in all vaccinated patients. In a recent study by Schroeder et al., the ^18^F-FDG-positive axillary lymph nodes were found in four out of 54 patients subjected to COVID-19 vaccination performed at the median time of 10–13 days earlier (15). This observation may cause even more uncertainty of the PET/CT image interpretation. Recently, authors from Israel have reported a much higher incidence of the vaccination-related axillary lymphadenopathy: 36.4% after the first vaccine dose and as much as 53.9% after the booster dose (16). It must be emphasized, however, that these results refer to the mRNA vaccine (Pfizer BNT162b2), not the viral vector vaccine, as in the presented case. If the vaccination and the PET/CT are to be performed in a short interval, we recommend to schedule the PET/CT before the COVID-19 vaccination. This may not always be feasible, especially if the patient undergoes an oncological treatment with a strict protocol. In such a situation, a delayed PET/CT scan would be a preferable solution. Considering the optimal time of PET/CT after vaccination, no firm data are available. From our experience, a great majority of patients who present the sign of increased axillary ^18^F-FDG accumulation received their vaccine in the recent 10 days. In rare cases, however, we have seen this sign even more than 4 weeks after the injection and this observation is supported by other authors (5). It is noteworthy that a prolonged nodal hypermetabolism is more likely to be found after the booster dose of a mRNA vaccine (16). Therefore, we recommend performing the PET/CT imaging *ca*. 4 weeks after the vaccination if the treatment protocol allows that. In any case, the physician responsible for reporting the PET/CT scan must be aware of the vaccination date.

## Conclusion

This case report presents possible misinterpretation of PET/CT images caused by a recent COVID-19 vaccination. To avoid distress of the patient and unnecessary oncological diagnostics to verify the findings, we recommend avoiding scheduling PET/CT shortly after vaccination.

## Data Availability Statement

The raw data supporting the conclusions of this article will be made available by the authors, without undue reservation.

## Author Contributions

RC wrote the manuscript. JS performed image acquisition and prepared images. JM prepared clinical data and the time-line and reviewed the manuscript. MR reviewed the manuscript. All authors contributed to the article and approved the submitted version.

## Conflict of Interest

The authors declare that the research was conducted in the absence of any commercial or financial relationships that could be construed as a potential conflict of interest.

## Publisher’s Note

All claims expressed in this article are solely those of the authors and do not necessarily represent those of their affiliated organizations, or those of the publisher, the editors and the reviewers. Any product that may be evaluated in this article, or claim that may be made by its manufacturer, is not guaranteed or endorsed by the publisher.
